# Affective components in promoting physical activity: A randomized controlled trial of message framing

**DOI:** 10.3389/fpsyg.2022.968109

**Published:** 2022-09-12

**Authors:** Valentina Carfora, Marco Biella, Patrizia Catellani

**Affiliations:** ^1^Department of Psychology, Catholic University of the Sacred Heart, Milan, Italy; ^2^Heidelberg University, Heidelberg, Baden-Württemberg, Germany

**Keywords:** message framing, message, physical activity, affective attitude, anticipated affective reactions

## Abstract

Although the study of the affective components involved in predicting physical activity is spreading faster and faster, there is a lack of studies testing their role when promoting physical activity through message interventions. In the present study, we considered these components by focusing on how anticipated affective reactions and emotional processing of the messages influence receivers’ affective attitude toward physical activity, concurrent behavior, and future intention. A sample of 250 participants was involved in an intervention relying on prefactual (i.e., “If … then…”) messages promoting physical activity. All messages were sent through a research app and were focused on the expected consequences of exercising (or not exercising). Four experimental conditions involving messages differing as to their outcome sensitivity framing (i.e., gain, non-loss, non-gain, and loss) were compared to a control condition. Results showed that reading gain and non-gain messages enhanced the positive affective attitude toward physical activity, compared to control. Enhanced affective attitude after the intervention increased, in turn, self-reported physical activity and future intention. Interestingly, gain messages were even more persuasive for people with a low level of positive anticipated affective reactions. Furthermore, their effectiveness was especially attributable to the elicitation of hope in receivers. Discussion focuses on the advantages of considering affective components and their implications when promoting physical activity.

## Introduction

Extensive literature shows that regular physical activity helps reduce the risk of diverse aversive health outcomes, stress, and even the risk of all-cause mortality to 31–39% (Haskell, 2009; Arem et al., 2015). Furthermore, regular physical activity increases emotional resilience, well-being, and quality of life (Childs and de Wit, 2014; Marquez et al., 2020). Besides health-related benefits, physical activity brings economic advantages in terms of social welfare, as the worldwide economic cost of physical inactivity is conservatively estimated to be around $53.8 billion per year by a recent “global analysis” (Ding et al., 2016). Therefore, not surprisingly, physical activity is recommended as routine practice. Specifically, the health guidelines recommend adults at least 150–300 min of moderate-intensity aerobic physical activity; at least 75–150 min of vigorous-intensity aerobic physical activity; or an equivalent combination of moderate-and vigorous-intensity activity throughout the week (Piercy et al., 2018). However, on average the general population fails to maintain the required level of physical activity (Haskell, 2009), paving the way for a thorough investigation of the psychological mechanisms responsible for such a failure. These mechanisms are potential points of attack to circumvent the setback of physical inactivity. In this regard, past research suggests that guidelines and recommendations alone are unlikely to produce the increase in physical activity needed to achieve the desired health outcomes (Milton et al., 2020). This makes the investigation of how to change people’s attitudes toward physical activity even more warranted.

Psychological research is already engaged in investigating viable ways to promote physical activity through persuasive messages aimed at increasing a positive attitude toward it (Gallagher and Updegraff, 2012; Carfora and Catellani, 2021; Catellani et al., 2021). The present paper builds upon this corpus of research to investigate how persuasive messaging interventions exert their influence in the context of physical activity by inducing peoples’ attitude change (Petty and Cacioppo, 1986; Eagly and Chaiken, 1993; Petty and Briñol, 2015). We tested the effectiveness of an intervention consisting of sending daily messages *via* an app^1^ about the consequences of engaging (or not) in physical activity on a regular basis. In this intervention, we employed prefactual (i.e., “If… then”) messages (Carfora and Catellani, 2021) and compared the effectiveness of four message frames, differing in how they presented the consequences of doing (or not) physical activity. Following the self-regulatory framework for message framing (Cesario et al., 2013), we referred to the model of outcome sensitivities and made a distinction among gain, non-loss, non-gain, and loss messages. We tested whether these differently framed messages would differently influence participants’ attitudes and, in turn, self-reported behaviors and future intention to do physical activity.

Several researchers have suggested that emotions play a crucial role in influencing attitude toward physical activity (Gross and D’ambrosio, 2004; Dillard and Nabi, 2006; Peters et al., 2006; Kühne and Schemer, 2015). We therefore expected that the affective components would be crucial in explaining and triggering attitude change toward engaging in physical activity. Following the Affect and Health Behavior Framework (AHBF; Williams and Evans, 2014), the affective components related to physical activity can be classified into four main classes, based on the relationship between the component and specific behaviors. These classes are the following: (1) affective response, which is related to the feelings and perceptions experienced immediately after or during the target behavior; (2) incidental affect, which refers to the feelings and perceptions experienced during the day when physical activity is carried out; (3) affect processing, which represents the (potentially automatic) cognitive processing of thoughts and affective responses related to previous or future instances of physical activity; and (4) affectively charged motivation, which relates to the motivational states linked to past physical activity. Focusing on this framework, we expected affect processing to be a key dimension in explaining the effectiveness of our messaging intervention. Specifically, we evaluated to what extent the four message frames would be effective as a function of the receivers’ anticipated affective reactions toward physical activity, as well as their cognitive and emotional processing of it.

## Theoretical background

The main objective of the present research was to promote regular physical activity through an intervention based on persuasive messages. Specifically, we aimed at investigating how messages can trigger more positive attitudes toward physical activity, and in turn, increase self-reported behavior and future intention to exercise. We started from the literature related to the effectiveness of persuasive messages in the domain of preventive health behaviors (e.g., Armanasco et al., 2017). Past research has widely shown that, under certain conditions, persuasive communication can induce attitude change and that the change in attitude may in turn lead to a change in behavior (e.g., Petty and Cacioppo, 1986; Ajzen, 1991; Eagly and Chaiken, 1993). To change attitude, persuasive communication often focuses on the most salient outcomes of the behavior in question. Consistent with Fishbein’s (1967a,b) summative model of attitudes, a persuasive message can attempt to change attitudes by modifying the perceived likelihood of different outcomes, modifying the perceived evaluation of different outcomes, or introducing new salient outcomes. This approach assumes that appropriate persuasive messages will produce changes in receivers’ attitudes that will impact intention and behavior. Based on this theoretical framework, in this study, we centered our messages on informing receivers about the possible outcome of doing (or not) physical activity using a prefactual (i.e., “If… then…”) formulation. Prefactual communication consists of presenting hypothetical future outcomes of possible actions (e.g., “If I take action X, it will lead to outcome Y”), and has been shown to be effective in influencing receivers’ attitudes, intentions, and behaviors (e.g., Bertolotti et al., 2020; Carfora et al., 2020).

Within the domain of studies related to physical activity, diverse empirical fundamentals have indicated that the enhancement of positive affective variables (such as those related to the expectation of enjoyment and pleasure) is more likely to facilitate physical activity than the enhancement of positive cognitive variables (such as those related to health and well-being expectations) (e.g., Conner et al., 2011; Nielsen et al., 2014). Consistently, a recent meta-analysis has confirmed that positive affective variables mediate the relationship between intervention and outcomes in terms of physical activity (Chen et al., 2020). Affective attitude has also been found to be a stronger predictor of several health-related behaviors (such as engaging in physical activity, exercising, consuming fruit and vegetables, and following a low-fat diet; Conner et al., 2015) compared to cognitive attitude, and its impact on behavior is independent of the intention to perform a specific behavior. Based on this evidence, we expected that a change in receivers’ affective attitudes would have positive effects on the improvement of physical activity.

Some researchers have shown that the persuasive effect of recommendation messages depends on how the messages are framed (Davis, 1995; Chong and Druckman, 2007; Spence and Pidgeon, 2010). Message framing refers to the evidence that decision-makers respond differently to diverse but objectively equivalent information (Kühberger, 1998, p. 150), which stresses either the positive or the negative consequences of a behavior (e.g., Rothman et al., 2008). In the context of the promotion of physical activity, prefactual messages can for example either emphasize the positive outcomes of exercising (positively-framed messages) or the negative outcomes of not exercising (negatively-framed messages). Existing literature supports the notion that positively-framed messages are slightly more effective in promoting physical activity (Jones et al., 2003; van’t Riet et al., 2010; Kozak et al., 2013; Williamson et al., 2020), but the bigger picture is far more complex and nuanced for at least two reasons.

First, positively- and negatively-framed messages can be further classified by adding a reference to the achievement or non-achievement of gains or losses (Cesario et al., 2013). This means that a positively-framed message can be formulated either as a *gain message*, which focuses on the positive consequences achieved by doing physical activty, or as a *non-loss message*, which focuses on avoiding the negative consequences deriving from low physical activity (Cesario et al., 2013). Similarly, a negatively-framed message can be framed both as a *non-gain message*, which emphasizes the missed positive consequences of engaging in physical activity, and a *loss message*, which emphasizes the negative consequences of not engaging in physical activity.

Comparing the effects of these four message frames is a theoretically sound way to create persuasive message-based interventions. So far, the possibly diverse effect of these frames has not been investigated in the domain of the promotion of physical activity. A notable exception is the studies by Carfora and Catellani (2021) and Catellani et al. (2021), who however only focused on the short-term effects of a single exposure to differently framed messages. Besides, those studies were conducted during the period of social isolation due to COVID-19, and for this reason they only focused on the promotion of exercising at home. In the current research, we sent messages over a longer period (15 days) and we addressed physical activity at large, indoor and outdoor.

Second, different scholars have suggested that more substantial effects of message framing might be observed by adopting an analysis which does not focus only on its direct effects on future action, but which allows understanding the process through which frames influence both cognitive and affective responses, which, in turn, may influence attitudinal and behavioral outcomes (Nabi et al., 2020). Thus, research on message framing can greatly benefit from the adoption of a more in-depth examination of the moderators that may augment or attenuate the effects of the messages, as well as the mediators that can intervene between frame exposure and persuasive effect.

Within the literature on the promotion of physical activity, moderated analyses revealed subgroups of participants who appeared to be especially receptive to gain or loss messages. Summarizing these studies, gain-framed messages are especially effective for encouraging physical activity among older adults, people with a goal achievement orientation (i.e., promotion oriented), and obese women (e.g., Latimer et al., 2008; Kozak et al., 2013; Li et al., 2014). So far, no scholars have studied which variables may moderate the persuasive effect of the four message frames on attitudes. Especially, none of them has considered the moderating effects of affective components. Thus, in this study we decided to focus on one of the most important affective predictors of physical activity, that is, the receivers’ anticipated affective reactions, namely, the anticipation of positive and negative emotions and feelings expected to be experienced during or after engaging (or failing to engage) in physical activity. Indeed, anticipated affective reactions are activated when an individual envisages experiencing certain emotions, cognitively projecting them into the future. They involve affectively-laden beliefs of how different situations are likely to influence emotions (Conner et al., 2016).

Leveraging several studies showing that the affective reactions sustain positive attitudes toward physical activity (e.g., Stevens et al., 2019), we expected that anticipated affective reactions would moderate the effect of prefactual messages. Given that anticipated affective reactions of future success and failure are a type of outcome expectancy thought to play a role in health behavior (e.g., Bagozzi, 1992; Richard et al., 1996; Bagozzi and Pieters, 1998; Abraham and Sheeran, 2003) and that our prefactual messages focused on the expected outcome of engaging or not in physical activity, we expected their persuasive impact on affective attitude to depend on how people can anticipate the affective reactions they will have when either adhering or not adhering to the message recommendation. For example, we can expect those who do not anticipate positive affective reactions to exercising (e.g., feeling satisfied) to prefer messages that precisely inform them of the positive consequences of exercising (i.e., gain messages).

Concerning the possible effects of some mediators between the message framing and its persuasive effects, the few studies assessing them have referred to social cognitive variables, particularly attitudes and intentions. Recently, researchers are instead calling for the exploration of alternate determinants, such as affect and information processing; Jackson et al., 2017). However, there is still a surprising paucity of studies on how emotional responses elicited in the audience when reading messages on physical activity may mediate the effect of these messages. A recent meta-analysis by Nabi et al. (2020) showed that gain-framed messages induce positive emotions while loss-framed messages induce negative emotions. In turn, the experience of positive emotions enhances the persuasive effect of gain-framed messages. These findings suggest that the emotional reactions to the messages may offer a pathway through which gain- and loss-framed messages exert their persuasive influence. However, so far, no study has assessed whether the emotional responses differ not only as regards gain-and loss-framed messages, but also non-gain- and non-loss-framed messages. Moreover, in line with what was shown by past studies on the emotional processing of message framing (Carfora and Catellani, 2021; Catellani et al., 2021), we could expect emotions induced by the messages to mediate the relationship between the intervention condition and participants’ attitudes after message exposure. Finally, we evaluated if the effect of emotional message processing was moderated by receivers’ prior anticipation of the positive and negative emotions they would experience in the future as a consequence of doing or not doing physical activity on a regular basis.

### The present study

Based on prior findings that the persuasive effect of health recommendation depends on how the messages are framed (Davis, 1995; Chong and Druckman, 2007; Spence and Pidgeon, 2010), in this study, we compared the effectiveness of gain, non-loss, non-gain, and loss messages. Given that the possibly different impact of these message frames has been little investigated in the domain of physical activity (except for Carfora and Catellani, 2021 and Catellani et al., 2021), we first tried to answer the following general research question.

*Research Question 1 **(RQ1)***: Do gain, non-loss, non-gain, and loss messages differently influence affective and cognitive attitudes toward physical activity?

Regardless of how messages were framed, we tested whether their prefactual formulation would stimulate a positive change in participants’ evaluation of the consequences of doing (or not doing) physical activity. Exposure to prefactual messages triggers prefactual thinking, that is future-directed imagination which induces an expectation about how a given outcome would change by altering its antecedent conditions (e.g., Bertolotti et al., 2020; Carfora et al., 2020). We therefore expected that prefactual messages would change participants’ evaluations of the consequences of physical activity or inactivity. We hypothesized that:

*Hypothesis 1 **(H1)***: Prefactual messages increase people’s positive cognitive (**H1a**) and affective attitudes (**H1b**) toward doing physical activity.

Moreover, past research showed that changing affective attitude toward physical activity enhances physical activity levels more than changing cognitive attitude (e.g., Nielsen et al., 2014; Chen et al., 2020). Thus, we hypothesized that:

*Hypothesis 2 **(H2)***: Receivers’ change in affective attitude enhances their physical activity (**H2a**) and intention to exercise on a regular basis (**H2b**).

We also considered the moderating role of anticipated affective reactions toward doing (or not doing) physical activity. In line with what was shown by several studies suggesting that anticipated affective reactions predict positive attitudes toward doing physical activity (e.g., Stevens et al., 2019), we expected that anticipated affective reactions would moderate the effect of prefactual messages. However, the literature available did not allow us to advance clear hypotheses on the interaction between each prefactual frame and prior positive and/or negative anticipated affective reactions. Therefore, we limited this study to observing these interactions and answering the research question stated below.

*Research Question 2*: To what extent do positive and negative anticipated affective reactions moderate the mediating effect of receivers’ attitudes?

Another aim of our study was to test whether the persuasive effect of our message intervention would be mediated by the emotional reactions to the messages. In doing this, we referred to prior evidence that gain-framed messages induce positive emotions while loss-framed messages induce negative emotions, and thus we hypothesized that:

*Hypothesis 3 **(H3)***: Gain messages induce more positive emotions (**H3a**) and less negative emotions (**H3b**) than loss messages.

However, given the scarcity of previous studies on the emotional reactions toward non-loss and non-gain messages we did not formulate any specific hypothesis regarding the emotional reactions to them, but only a research question.

*Research Question 3*: Do non-loss and non-gain framed messages differently influence the emotional processing of the messages?

Moreover, leveraging past studies on the mediational role of the emotional evaluations activated by differently framed messages (Carfora and Catellani, 2021; Catellani et al., 2021), we wondered whether emotions induced by the messages would mediate the relationship between the intervention condition and participants’ attitudes after message exposure.

*Research Question 4*: Does emotional message evaluation mediate the impact of message framing on receivers’ attitudes?

Finally, we evaluated if the aforementioned mediating effect of the emotional message processing was moderated by receivers’ anticipated affective reactions.

*Research Question 5 **(RQ5)***: To what extent do positive and negative anticipated affective reactions moderate the effects of gain-, non-loss, non-gain-, and loss-framed messages on message processing and then attitudes?

## Materials and methods

### Participants

Ethical approval for this study was provided by the Catholic University of the Sacred Heart (Milan) and the Scientific Committee of the Istituti Clinici Scientifici Maugeri (Pavia). Using GPower 3.1, we conducted a sample size estimation considering an *Effect size f* = 0.25. With an *alpha* = 0.05, *power* = 0.90, *number of groups* = 5 (message conditions), *number of measurements* = 8 (4 measure at 2 time points), *correlations among repeated measure* = 0.50, and *p* = 0.05, the projected sample size needed was approximately N = 55, and specifically about 10 participants per group.

Then, we conducted a second sample size estimation for the moderation analysis aimed at testing the different effects of message framing depending on receivers’ anticipated affective reactions. Considering an *Effect size* f = 0.25, with an *alpha* = 0.05, *power* = 0.90, *number of predictors* = 21, and *p* = 0.05, the projected sample size needed for the regressions was approximately N = 124, and specifically about 25 participants per group. To ensure this sample size despite any dropouts during the intervention phase, we aimed to at least double the number of participants needed.

In February 2022, we invited 250 Italian individuals to participate in a university study through Prolific (https://www.prolific.com), a platform for online subject recruitment designed for research. On the Prolific webpage, the study was advertised as research on lifestyle, about a total of 40 min in length, and distributed in 15 days. Participants were informed that they were recruited for a 15-day long research and about the expected payment for participation in the study. All participants had to be residents of Italy and have a Prolific record of at least 75% satisfactorily completed experiments. We looked for a sample of participants who needed to increase their level of physical activity to reach the recommended level. So, we only accepted participants who were doing less than 150 min of moderate aerobic activity per week or the equivalent of 75 min of vigorous activity (Bull et al., 2020).

After accessing the study on Prolific, participants provided informed consent through a questionnaire implemented on the Qualtrics platform. In the same questionnaire, participants found the instructions to access the PsyMe app using an anonymous alphanumerical code, and to correctly participate in the study using the app. The alphanumerical codes were generated using an automatic randomization sequence, through which participants were randomly assigned to one of the five experimental conditions of the study (gain, non-loss, non-gain, loss messages, and a control condition).

Using the PsyMe app, participants accessed the Time 1 (T1) questionnaire. At the beginning of the questionnaire, we provided participants with instructions to properly fill out the online questionnaire. Then, participants received one message per day for 15 days, except for participants assigned to the control condition. At the end of the 15-day intervention period, all participants completed the Time 2 (T2) questionnaire. A control question to verify if participants’ replies were reliable was included in both questionnaires. Finally, participants received feedback on the aims of the study. Those completing the entire research were paid £5.00.

**Figure 1:**
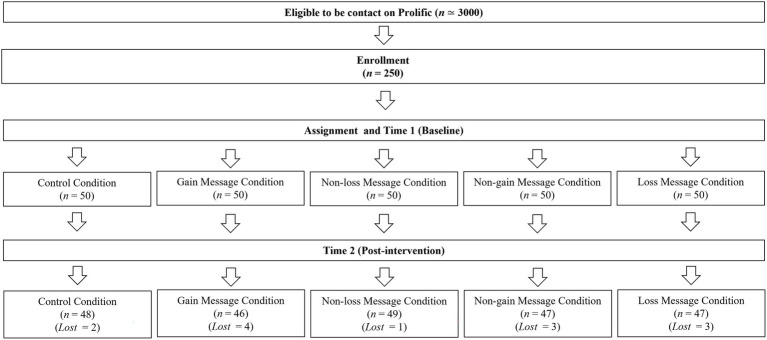
Flow chart of participants’ recruitment.

Figure 1 shows the flow of participants during the study. At T1, 250 participants accessed the PsyMe app and correctly completed the questionnaire (136 females, 114 males; age range 18–65 years, *mean* age = 29.24, *SD* age = 8.66). After the intervention, at T2, 237 participants correctly filled in the second questionnaire and were retained as the final sample of our study (129 females, 108 males; age range 18–65 years, M age = 29.07, SD age = 8.63).

### Pre-test measures

The questionnaire at T1 included several measures. Below, we report the relevant measures for the present paper.

*Cognitive attitude toward physical activity* was measured with five items rated on a semantic differential scale ranging from “1” to “7” (e.g., “Exercising is … useless – useful”; Caso et al., 2021). Higher values indicated a more positive cognitive attitude toward exercising. Cronbach’s alpha was 0.87.

*Affective attitude toward physical activity* was measured with four items rated on a semantic differential scale ranging from “1” to “7” (e.g., “Exercising is … unpleasant – pleasant”; Caso et al., 2021). Higher values indicated a more positive affective attitude toward exercising. Cronbach’s alpha was 0.87.

*Positive anticipated affective reactions* were measured with six items on a seven-point Likert scale (e.g., “If I exercise regularly … I will be proud of myself”; Catellani et al., 2021). Higher values indicated higher positive anticipated affective reactions toward exercising. Cronbach’s alpha was 0.89.

*Negative anticipated affective reactions* were measured with six items on a seven-point Likert scale (e.g., “If I do not exercise regularly … I will regret it”; Catellani et al., 2021). Higher values indicated higher negative anticipated affective reactions toward not exercising. Cronbach’s alpha was 0.86.

*Frequency of physical activity* was measured with 2 items regarding how often participants engaged in physical activity away from home and at home: “On average, how many times a week do you engage in a moderate or intense physical activity outdoor - e.g. fast walking, climbing stairs, cycling, swimming, going to the gym, going for a run, etc.?”; “On average how many times a week do you exercise at home?” (Carfora and Catellani, 2021). Answers were given on a seven-point Likert scale, from never (1) to every day (7). Higher scores indicated a higher frequency of physical activity.

*Intention toward doing physical activity* was assessed with three items on a seven-point Likert scale (e.g., “In the next month, I intend to exercise regularly”; completely disagree (1) – completely agree (7); Catellani et al., 2021). Higher scores indicated a greater intention to exercise regularly. Cronbach’s alpha was 0.96.

Finally, we asked participants socio-demographic information including age, sex, level of education, and marital status.

### Message intervention

During the 14-day intervention (between T1 and T2), all participants (except those in the control condition) received daily persuasive messages *via* the PsyMe app. Thus,14 messages were sent in each condition. The full list of messages is reported in Supplementary Table 1. All messages described the physical, mental, and social positive consequences of doing physical activity. Also, they were formulated as prefactuals, namely, as a conditional proposition about an action-outcome linkage that may (or may not) occur in the future (e.g., “If you take action X, it will lead to outcome Y”; Carfora and Catellani, 2021). Participants in the *gain message condition* received messages focused on the positive consequences of doing physical activity on a regular basis (e.g., “If you exercise regularly, you will improve your fitness”). Participants in the *non-loss message condition* received messages on the avoidance of negative consequences of doing physical activity on a regular basis (e.g., “If you will exercise regularly, you will avoid worsening your fitness.”). Participants in the *non-gain message condition* received messages on the loss of positive consequences of not doing physical activity on a regular basis (e.g., “If you do not exercise regularly, you will lose the chance to improve your fitness”). Finally, participants in the *loss message condition* received messages on the negative consequences of not doing physical activity on a regular basis (e.g., “If you do not exercise regularly, you will lose the chance to improve your fitness”).

### Post-test measures

After the 15-day intervention, participants were administered a questionnaire aimed at measuring the dimensions described below.

*Message reading frequency* was obtained through the PsyMe app, which keeps track of the reception of the messages.

*Manipulation check* was conducted asking the participants to select among four messages the one which was most similar to the messages they received for 14 days through the PsyMe app.

*Message involvement* was measured with six items asking participants to state how involved they had been in the messages (e.g., “The message was very interesting”; adapted from Carfora and Catellani, 2021). Answers were given on a 7-point Likert scale, from (1) “strongly disagree” to (7) “strongly agree.” Higher values indicated a higher participant’s positive evaluation of the messages. Cronbach’s alpha was 0.88.

*Message trust* was assessed with three items on a 7-point Likert scale (e.g., “Do you think the information presented in the message is reliable? (1) Not at all – (7) Extremely”; Carfora and Catellani, 2021). Higher values indicated a higher trust in the messages. Cronbach’s alpha was 0.92.

*Systematic processing* was measured with five items, asking participants to state how deeply they had processed the information presented in the messages (e.g., “I tried to think about the importance of the information for my daily life”; Carfora and Catellani, 2021). Answers were given on a 7-point Likert scale, from (1) “strongly disagree” to (7) “strongly agree.” Higher values indicated a deeper processing of the messages. Cronbach’s alpha was 0.84.

*Message-induced fear* was measured with six items pertaining to the degree to which reading messages had made participants feel fearful (e.g., “To what extent reading these messages did you feel scared?”; adapted from Carfora and Catellani, 2021). Answers were given on a 7-point Likert scale, from (1) “not at all” to (7) “completely.” Higher values indicated a higher participant’s fear after reading the messages. Cronbach’s alpha was 0.81.

*Message-induced anger* was measured with three items related to how irritated the receivers felt after reading the messages (e.g., “To what extent reading these messages did you feel irritated?”; adapted from Carfora and Catellani, 2021). Answers were given on a 7-point Likert scale, from (1) “not at all” to (7) “completely.” Higher values indicated a higher participant’s anger after reading the messages. Cronbach’s alpha was 0.91.

*Message-induced anxiety* was assessed with three items related to how anxious the recipients felt after reading the messages (e.g., “To what extent reading these messages did you feel anxious?”; adapted from Carfora and Catellani, 2021). Answers were given on a 7-point Likert scale, from (1) “not at all” to (7) “completely.” Higher values indicated a higher participant’s anger after reading the messages. Cronbach’s alpha was 0.80.

*Message-induced hope* was investigated using three items related to how hopeful the participants felt after reading the messages (e.g., “To what extent reading these messages did you feel hopeful?”; adapted from Carfora and Catellani, 2021). Answers were given on a 7-point Likert scale, from (1) “not at all” to (7) “completely.” Higher values indicated a higher participant’s anger after reading the messages. Cronbach’s alpha was 0.86.

*Message-induced calm* was assessed using three items related to how calm the participants felt after reading the messages (e.g., “To what extent reading these messages did you feel calm?”; adapted from Carfora and Catellani, 2021). Answers were given on a 7-point Likert scale, from (1) “not at all” to (7) “completely.” Higher values indicated a higher participant’s anger after reading the messages. Cronbach’s alpha was 0.89.

Finally, we again measured receivers’ cognitive and affective attitudes, frequency of behavior, and future intention, with the same scale used at Time 1. Cronbach’s alpha was 0.84 for cognitive attitude 0.88 for affective attitude and 0.95 for intention.

### Data analysis

All analyses were conducted in SPSS 24. In preliminary analyses, to check if randomization was successful, we used a multivariate analysis of variance MANOVA, testing if there were differences among conditions on affective attitude, cognitive attitude, positive anticipated affective reactions, negative anticipated affective reactions, intention, frequency of physical activity, and age at T1. Chi-square was used to check any significant differences in gender, marital status, and level of education across conditions. Using an ANOVA, we also checked if message reading frequency was influenced by message frame. Next, with a Chi-square we did a manipulation check by controlling whether participants correctly identified the frame of the messages they received during the intervention. We also ran another MANOVA to verify if there were differences in the cognitive elaboration of the messages (i.e., message involvement, message trust, and systematic processing) in the four conditions. With a further MANOVA, we analyzed whether dropouts were explained by the participants’ levels in study variables at T1.

In the main analyses, we first answered our **RQ1** and tested our **H1a** and **H1b**. We compared the impact of message framing on participants’ cognitive and affective attitudes toward physical activity. Thus, we conducted a 5 (four message conditions and control condition) X 2 (T1 vs. T2) ANOVA with cognitive attitude and affective attitude as dependent variables, with repeated measures on the last factor (Table 1). Then, we ran a mediation analysis using bootstrapping in SPSS (PROCESS macro for SPSS), where the indirect effects were considered significant if bootstrapped 95% confidence intervals (CI) did not include zero. In these analyses, we tested whether affective attitude at T2 explained participants’ physical activity during the message intervention and future intention at T2 (**H2a** and **H2b**). Specifically, we ran two mediation analyses (Model 4 of the PROCESS macro for SPSS; Hayes and Preacher, 2013). In the first mediation analysis, message condition (control = 0; gain message = 1; non-loss message = 2; non-gain message = 3; loss message = 4) was the independent variable, physical activity at T2 was the dependent variable, affective attitude at T2 was the mediator, and affective attitude and intention both at T1 were covariates.

**Table 1:** Results of the ANOVA involving affective and cognitive attitude toward physical activity.

Predictor	*df*	*F*	*p*	*ηp^2^*
**Affective attitude toward doing physical activity**				
Intercept	1, 232	3297.36	0.001	0.93
Time	1, 232	47.29	0.001	0.17
Condition	4, 232	0.98	0.42	0.02
Time x condition	4, 232	3.80	0.005	0.06
**Cognitive attitude toward doing physical activity**				
Intercept	1, 232	14764.90	0.001	0.96
Time	1, 232	1.34	0.25	0.01
Condition	4, 232	0.63	0.64	0.11
Time x condition	4, 232	0.21	0.93	0.01

Next, we verified whether the mediating effect of affective attitude at T2 on self-reported physical activity and future intention (both at T2) was determined by participants’ affective reactions at T1 (**RQ2**) by conducting moderated mediation models (Model 11 of the PROCESS macro for SPSS; Hayes and Preacher, 2013). Precisely, we first ran a moderated mediation model (Model 11 of the PROCESS macro for SPSS; Hayes and Preacher, 2013) including message condition (gain = 1; non-loss = 2) as the independent variable, physical activity at T2 as the dependent variable, affective attitude at T2 as the mediator, positive and negative anticipated affective reactions at T1 as moderators, and affective attitude and past frequency of physical activity both at T1 as the covariates. Then, we ran a similar analysis considering future intention at T2 as the dependent variable and affective attitude and future intention at T1 as covariates.

To assess whether the emotional evaluation of the messages varied according to message framing (**RQ3** and **H4**), we carried out a MANOVA including message-induced anger, fear, anxiety, hope, and calm. Finally, we conducted the analyses related to our **RQ4** and **RQ5**. To compare the effects of the emotions elicited by gain and loss messages, we first ran a mediation model (Model 4 of the PROCESS macro for SPSS; Hayes and Preacher, 2013) including gain message condition (code 1) versus loss message condition (code 2) as the independent variable, affective attitude at T2 as the dependent variable, and affective attitude at T1 as the covariate. In this analysis, message-induced anger, fear, anxiety, calm, and hope were included as mediators. Then, we ran a moderated mediation model (Model 11 of the PROCESS macro for SPSS; Hayes and Preacher, 2013) including positive and negative anticipated affective reactions as moderators. To contrast the effects of the emotions elicited by the non-gain and loss messages, also considering the participants’ anticipated affective reactions at Time 1, again we ran a mediation model and then a moderated mediation model.

## Results

### Preliminary analyses

Table 2 reports the socio-demographic characteristics of the final sample. The sample was well balanced in terms of gender. However, most participants were single, young or young adults, and well educated.

**Table 2:** Demographics of the final study sample.

Characteristic	Percentage on the total sample
**Gender**	
Female	54.4%
Male	45.6%
**Age**	
Young (18–24 years)	36.0%
Young adults (25–34 years)	43.2%
Adults (35–54)	18.8%
Senior (55–65)	2.0%
**Education**	
Secondary school	2.0%
High school diploma	14.8%
University degree	83.2%
**Marital status**	
Single	71.6%
Married	11.2%
Cohabiting couple	15.2%
Separated/divorced	1.6%
Widow	0.4%

At the beginning of the intervention, participants had very positive cognitive attitudes toward exercising on a regular basis, while they had lower positive affective attitudes. This suggests that participants were more inclined to recognize that physical activity has concrete benefits than that physical activity has positive emotional consequences, such as pleasure and gratification. Anyhow, they anticipated positive emotions more than negative ones. In addition, participants had an average intention to do physical activity in the following month and a maximum weekly frequency of one training session. Table 3 reports the means and standard deviations of all study variables in the total sample and among conditions. Table 4 reports the correlations between these variables.

**Table 3:** Means and standard deviations of measured variables in each message condition.

Variables	Control condition (*n* = 48)	Gain message condition (*n* = 46)		Non-loss message condition (n = 49)		Non-gain message condition (n = 46)		Loss message condition (n = 47)		Total (N = 237)		
	*M*	*SD*	*M*	*SD*	*M*	*SD*	*M*	*SD*	*M*	*SD*	*M*	*SD*
**Time 1**												
Cognitive attitude toward physical activity	6.30	0.70	6.39	0.71	6.16	0.92	6.27	0.96	6.16	1.22	6.26	0.92
Affective attitude toward physical activity	4.19	1.20	4.34	1.29	4.29	1.32	4.40	1.27	4.28	1.30	4.30	1.26
Positive anticipated affective reactions	4.89	0.87	5.00	1.09	4.77	1.06	5.01	0.88	5.03	1.02	4.94	0.97
Negative anticipated affective reactions	3.86	1.10	3.50	1.20	3.60	1.19	3.67	1.23	3.82	1.22	3.69	1.19
Frequency of physical activity	3.64	1.13	3.78	1.13	3.59	1.15	3.72	1.06	3.59	1.13	3.67	1.12
Intention	4.14	1.67	4.68	1.87	4.31	1.67	4.78	1.50	4.18	1.71	4.41	1.69
**Time 2**												
Systematic processing	-	-	49.96	1.13	4.93	1.17	4.63	1.13	4.76	1.21	4.82	1.16
Message trust	-	-	5.58	1.04	5.54	1.04	5.41	0.89	5.47	1.09	5.50	1.02
Message involvement	-	-	4.81	1.18	4.74	1.17	4.54	1.34	4.38	1.43	4.62	1.29
Messgae-induced anger	-	-	1.44	0.61	1.77	0.86	1.90	1.06	2.01	0.88	1.79	0.89
Messgae-induced fear	-	-	1.30	0.62	1.43	0.57	1.61	0.65	1.84	0.79	1.55	0.69
Messgae-induced anxiety	-	-	2.91	0.97	1.70	0.58	1.80	0.71	2.09	0.85	1.78	0.72
Messgae-induced hope	-	-	3.02	0.90	2.49	0.95	2.50	0.79	2.27	0.82	2.54	0.91
Messgae-induced calm	-	-	6.45	0.68	2.89	0.84	2.83	0.85	2.46	0.98	2.80	0.91
Cognitive attitude toward physical activity	6.28	1.00	4.98	1.15	6.22	0.89	6.35	0.67	6.28	0.96	6.31	0.085
Affective attitude toward physical activity	4.38	1.30	6.16	0.92	4.62	1.26	4.88	1.16	4.37	1.34	4.64	1.26
Frequency of physical activity	3.81	1.62	4.29	1.32	3.86	1.96	4.00	2.15	3.96	1.72	3.98	1.91
Future intention	4.56	1.68	5.16	1.41	4.83	1.53	4.83	1.67	4.28	1.78	4.64	1.64

*M* = Mean; *SD* = Standard Deviation.

**Table 4:** Correlations among study variables before and after the message intervention.

	1	2	3	4	5	6	7	8	9	10	11
1. Cognitive attitude toward physical activity at T1	-										
2. Affective attitude toward physical activity at T1	0.46^***^	-									
3. Positive anticipated affective reactions at T1	0.39^***^	0.59^***^	-								
4. Negative anticipated affective reactions at T1	0.24^***^	0.32^***^	0.49^***^	-							
5. Intention at T1	0.34^***^	0.55^***^	0.49^***^	0.44^***^	-						
6. Frequency of physical activity at T1	0.10^**^	0.42^***^	0.26^***^	0.28^***^	0.55^***^	-					
7. Cognitive attitude toward physical activity at T2	0.50***	0.35***	0.28^***^	0.22^***^	0.25^***^	0.07	-				
8. Affective attitude toward physical activity atT2	0.36***	0.80***	0.58^***^	0.29^***^	0.51^***^	0.35***	0.38^***^	-			
9. Positive anticipated affective reactions at T2	0.35***	0.50***	0.75^***^	0.44^***^	0.50^***^	0.26***	0.34^***^	0.56^***^	-		
10. Negative anticipated affective reactions at T2	0.26***	0.28***	0.43^***^	0.66^***^	0.42^***^	0.22***	0.31^***^	0.32^***^	0.55^***^	-	
11. Future intention at T2	0.32***	0.53***	0.51^***^	0.40^***^	0.79^***^	0.48***	0.30^***^	0.56^***^	0.57^***^	0.46^***^	-
12. Frequency of physical activity at T2	0.12**	0.37***	0.28***	0.25***	0.50***	0.68***	0.09*	0.35^***^	0.30^***^	0.24^***^	0.50^***^

*T1* = Time 1; *T2* = Time 2. All *p*-values are significant. ^*^p < 0.05; ^**^p < 0.01; ^***^p < 0.001.

Results did not show any significant main effect of message conditions (*p* = 0.56, *ηp^2^* = 0.03) on affective attitude, cognitive attitude, positive anticipated affective reactions, negative anticipated affective reactions, intention, frequency of physical activity, and age at T1. Chi-square also did not show any significant differences in gender, marital status, and level of education across conditions (all *p* > 0.11). This suggests that the randomization was adequate, with the five conditions being comparable to the baseline variables.

Then, we verified if participants in the experimental conditions had read our messages. 2.5% of participants read 11 out of 15 messages, 14% read 12/13 messages, and 82.5% read 14/15 of them. ANOVA results did not reveal any difference among conditions in message reading frequency (*p* = 0.21). Chi-square also showed significant differences in message frame identification (336.27, *p* = 0.001) across conditions, confirming that the difference among message frames was understood and recognized by participants. Multivariate results showed that there was no main effect of the condition (*F*(9,549) = 1.66 *p* = 0.71, *ηp^2^* > 0.01), indicating that the messages were all systematically processed and perceived as involving and credible.

Regarding dropouts (Figure 1), 13 participants dropped out at post-test (T2). Chi-square did not show any significant differences in dropouts (*p* = 0.77) across conditions. Moreover, the MANOVA analysis indicated that dropouts were not explained by affective attitude, cognitive attitude, positive anticipated affective reactions, negative anticipated affective reactions, intention, and frequency of physical activity at T1(*p* = 0.50). These results and the low rate of dropout in our 14-day intervention allowed us to assert that dropouts were not determined by conditions or prior participants’ levels on study variables.

### Effects of prefactual messages on cognitive and affective attitudes

We examined the impact of message intervention on participants’ cognitive and affective attitudes toward physical activity, evaluating if some differences in the four message conditions emerged. Results showed a strong main effect of time on affective attitude, as well as an interaction between message condition and time. Therefore, there were differences in the extent to which the four message conditions resulted in a more positive affective attitude in T2 as compared to T1. Specifically, at T2 participants in the gain (*M* = 6.16; *SD* = 0.92) and non-gain message conditions (*M* = 4.88; *SD* = 1.16) had a more positive affective attitude compared to participants in the loss (*M* = 4.37; *SD* = 1.03) and control message conditions (*M* = 4.38; *SD* = 1.30) (all *p* = 0.05). Participants’ cognitive attitudes did not change after the intervention. Thus, we did not include this variable in the subsequent analyses.

In sum, we answered our **RQ1** finding that gain and non-gain-framed messages were the most effective intervention to increase participants’ positive affective attitudes toward exercising on a regular basis. We also confirmed our **H1b**, related to the positive effects of prefactual messages on receivers’ affective attitudes toward physical activity, while we disconfirmed our **H1a**, related to the effect of prefactual messages on receivers’ cognitive attitudes.

### The mediating effect of affective attitude on self-reported behavior and future intention

We tested whether affective attitude at T2 explained participants’ physical activity during the message intervention (**H2a**) and future intention at T2 (**H2b**). The results of the two mediation analyses showed that the more participants in the gain and non-gain message conditions had a highly positive affective attitude toward physical activity, the more they did physical activity regularly during the intervention (*Ind. Effects:* gain message condition: 0.21; *95%CI*: 0.05, 0.49; non-gain message condition: 0.15; *95%CI*: 0.01, 0.44).

In the second mediation analysis, intention at T2 was the dependent variable and the other variables remained the same as above. Again, the more participants in the gain and non-gain message conditions had a highly positive affective attitude, the more they expressed their intention to do physical activity on a regular basis in the month after the intervention (*Ind. Effects:* gain message condition: 0.12; *95%CI*: 0.03, 0.29; non-gain message condition: 0.08; *95%CI*: 0.01, 0.21).

In both analyses, message conditions did not directly explain changes in behavior and intention. Thus, changes in affective attitude fully mediated the effect of the intervention on participants’ self-reported behavior and intention, supporting our **H2a** and **H2b**.

### The moderating effect of anticipated affective reactions at time 1 on affective attitude at time 2

To address our **RQ2**, we explored whether the mediating effect of affective attitude at T2 on self-reported physical activity and future intention (both at T2) was influenced by participants’ affective reactions at T1.

**Figure 2:**
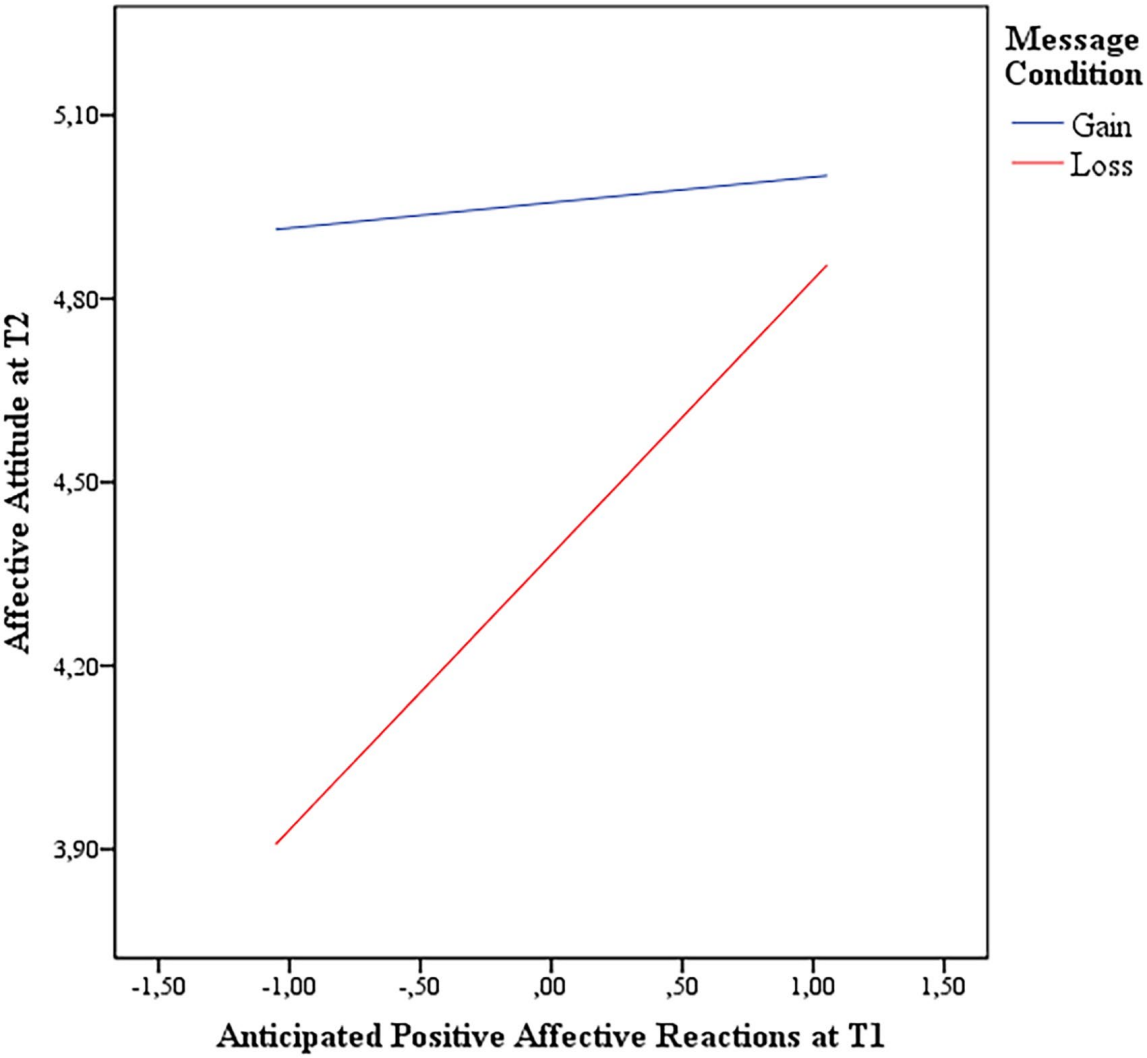
Affective attitude at time 2 in gain and loss message conditions at different levels of positive anticipated affective reactions.

The results of the moderated mediation analyses confirmed that participants in the gain message condition had a more positive affective attitude at T2 than those in the loss message condition (*B*: -0.66; *95%CI*: −0.91, −0.40). In addition, a significant interaction between positive anticipated affective reactions at T1 and affective attitude at T2 (*B*: 0.32; *95%CI*: 0.04, 0.59; Figure 2) showed that in the gain message condition affective attitude at T2 was higher than in the loss message condition, especially when positive anticipated affective reactions were low (*Ind. Effect* at low level of positive anticipated affective reactions = −1.00; *95%CI* = −1.32, −0.67; *Ind. Effect* at medium level of positive anticipated affective reactions = −0.57; *95%CI* = −0.80, −0.34). Also, affective attitude at T1 explained affective attitude at T2 (*B*: 0.67; *95%CI*: 0.55, 0.78). The frequency of physical activity at T2 was directly predicted by affective attitude at T2 (*B*: 0.67; *95%CI*: 0.55, 0.78). Indirect effects showed that participants’ in the gain condition with a low (−0.49; *95%CI*: −0.95, −0.07) or medium level of positive anticipated affective attitude at Time 1 had a more positive affective attitude at Time 2 and, in turn, a higher frequency of physical activity at T2. The index of the moderated mediation was 0.20 (*95%CI*: 0.02, 0.44). We repeated the above analyses to compare non-gain and loss messages, but we did not find any significant interaction between affective attitude and anticipated affective reactions. Finally, the same analyses considering future intention at T2 did not reveal significant interactions.

In sum, gain messages were more effective than loss messages in increasing affective attitude and then physical activity when participants had low anticipated positive affective reactions before message exposure.

### Effects of framing on the cognitive and emotional evaluation of the messages

Multivariate results showed a main effect of framing condition (*F*(15,534) = 2.11 *p* > 0.001, *ηp^2^* > 0.06). Univariate effects indicated that different frames generated different emotional processing in terms of message-induced anger (*F*(3,183) = 3.67, *p* > 0.01, *ηp^2^* > 0.06), fear (*F*(3,183) = 5.78, *p* > 0.01, *ηp^2^* > 0.09), anxiety (*F*(3,183) = 5.29, *p* > 0.002, *ηp^2^* > 0.08), hope (*F*(3,183) = 3.30 *p* > 0.07, *ηp^2^* > 0.06), and calm (*F*(3,183) = 3.26, *p* > 0.01, *ηp^2^* > 0.05). Pairwise comparisons (*p* > 0.05) indicated that gain messages were perceived as inducing less anger than non-gain and loss messages. Also, loss messages were perceived as scarier and more alarming than gain and non-loss messages. Finally, gain messages were perceived as more hopeful and calmer than loss messages.

Therefore, consistent with our **H3** gain messages induced more positive and less negative emotions than loss messages. As to our **RQ3** regarding emotions induced by non-loss and non-gain messages, we observed that non-gain messages induced more anger than gain messages, whereas non-loss messages induced less fear and anxiety than loss messages.

### The mediation of the emotional processing and the moderation of anticipated affective reactions

We finally tested whether the emotional processing of the messages mediated the significant effects of gain and non-gain messages on receivers’ affective attitude toward physical activity at T2 (**RQ4**), also based on positive and negative anticipated emotions at Time 1 (**RQ5**). Thus, we first carried out a first series of analyses to compare the gain and loss messages and a second series of analyses to compare non-gain and loss messages.

#### Gain versus loss messages

The findings of the mediation analysis confirmed that participants in the gain message condition had a more positive affective attitude at T2 than those in the loss message condition (*B*: −0.36; *95%CI*: −0.68, −0.05). In addition, participants’ positive affective attitude at T2 was predicted by message induced-fear (*B*: 0.33; *95%CI*: 0.01, 0.65) and message induced-hope (*B*: 0.40; *95%CI*: 0.16, 0.65), as well as by affective attitude at Time 1 (*B*: 0.70; *95%CI*: 0.59, 0.82). The indirect effects showed that only message-induced hope mediated the stronger effect of gain messages on affective attitude at T2, that is, gain messages were more effective than loss messages also because they stimulated more hope in the receivers (B: −25; *95%CI*: −0.49, −0.08).

As to message-induced fear, the outcomes of the moderated mediation analyses showed the presence of a significant interaction between positive and negative anticipated affective reactions (*B*: 0.33; *95%CI*: 0.01, 1.65), as well as a significant interaction among message conditions, positive anticipated affective reactions and negative anticipated affective reactions (*B*: −0.19; *95%CI*: −0.41, 0.01; Figure 2). Receivers with a low level of positive anticipated affective reactions and a high level of negative anticipated affective reactions (*B*: 0.92; *95%CI*: 0.08, 1.76) or a low level of negative anticipated affective reactions and a high level of positive anticipated affective reactions (*B*: 0.77; *95%CI*: 0.04, 1.51) were more scared by loss than gain messages. Instead, loss and gain messages were equally scary when people did anticipate both positive and negative anticipated affective reactions a lot (*B*: 0.35; *95%CI*:- 0.08, 0.79) or not at all (*B*: −0.40; *95%CI*: 0.06, −0.86). However, there were no moderated mediation effects, that is, the level of anticipated affective reactions at T1 did not impact affective attitudes at T2 *via* the receivers’ emotional processing.

In sum, gain messages were better than loss messages *per se*, and because they activated greater hope in the participants. Therefore, gain messages had both a higher positive effect on affective attitude at T2 and an indirect effect by inducing hope in receivers. Besides, overall a moderate level of fear determined a more positive affective evaluation of physical activity, regardless of whether it was promoted with a frame of gain or loss.

#### Non-gain versus loss messages

The findings of the mediation analyses confirmed that the non-gain messages were more effective than the loss messages (*B*: −0.34; *95%CI*: −0.64, −0.04). Besides the positive effects of affective attitude at Time 1 (*B*: 0.68; *95%CI*: 0.56, 0.81), affective attitude at T2 was only positively predicted by message induced-hope (*B*: 0.28; *95%CI*: 0.03, 0.53), which however did not mediate the effect of message condition (*B*: −0.05; *95%CI*: −0.18, 0.03).

**Figure 3:**
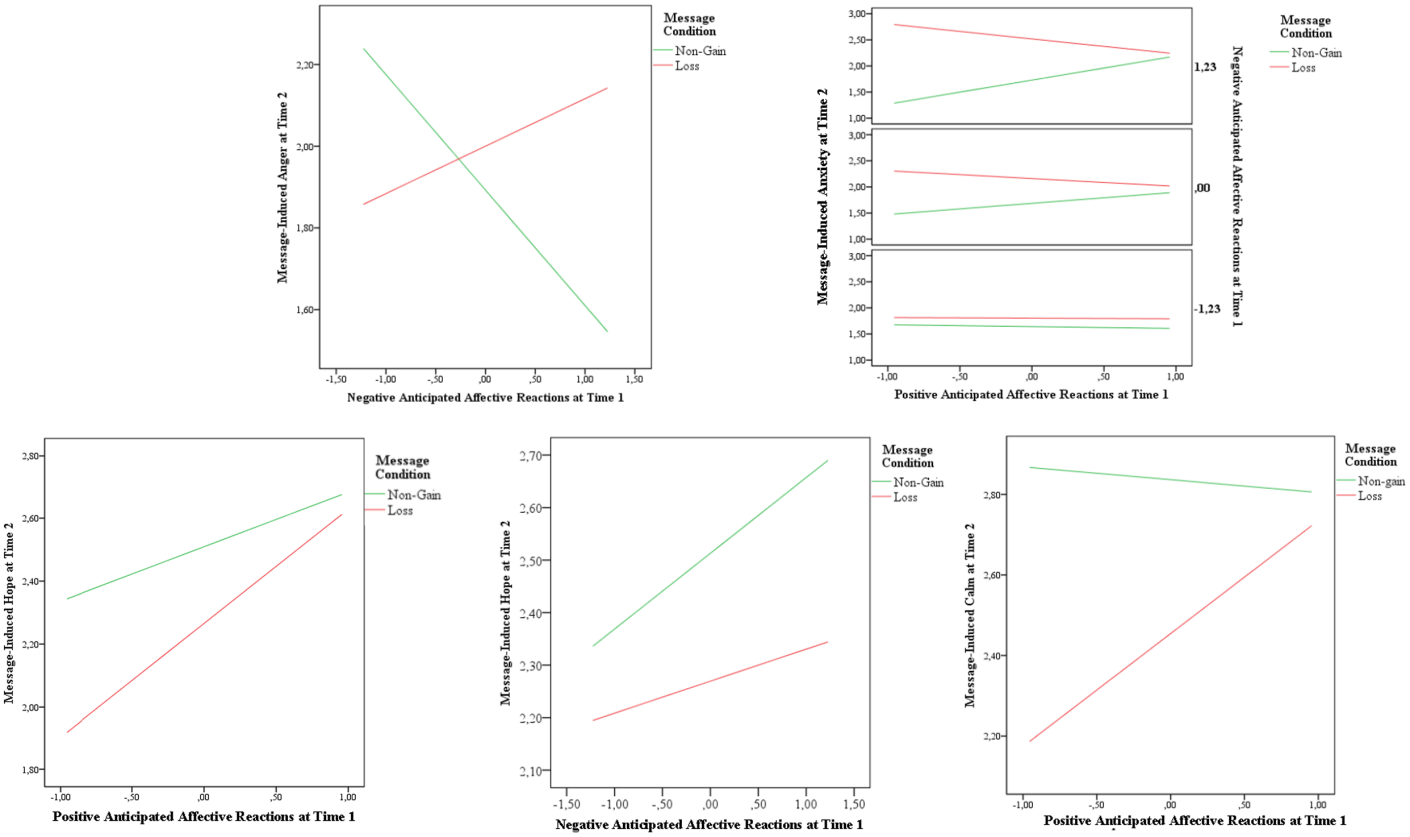
Message-induced emotions at time 2 in non-gain and loss message conditions at different levels of positive and negative anticipated affective reactions.

The moderated mediation outcomes added some evidence of significant interactions between positive and/or negative anticipated affective reactions and message conditions. In the case of message-induced anger, the message condition interacted with the receivers’ negative anticipated affective reactions (*B*: 0.49; *95%CI*: 0.08, 0.90; Figure 3), showing that when participants had high anticipation of the negative affective reactions they also perceived the loss message as more irritating than the non-gain message (*B*: 0.60; *95%CI*: 0.05, 1.14).

As for message-induced anxiety, there were significant interactions both between positive and negative anticipated affective reactions (*B*: 0.50; *95%CI*: 0.02, 0.97), and among message conditions, positive and negative anticipated affective reactions (*B*: −0.30; *95%CI*: −0.58, −0.02), showing that participants with inconsistency in anticipating their positive and negative affective reactions perceived the loss message as more alarming than the non-gain message especially when had a low positive anticipated affective reactions and high negative anticipated reactions (*B*: 1.50; *95%CI*: 0.40, 2.60; Figure 3).

Regarding message-induced hope, there was a significant interaction between message condition and positive anticipated affective reactions (*B*: 0.47; *95%CI*: 0.06, 0.89), as well as between message condition and negative anticipated affective reactions (*B*: −0.31; *95%CI*: −0.62, −0.01). Non-gain messages were perceived as more hopeful than the loss messages by participants with low positive anticipated affective reactions *B*: -0.30; *95%CI*: 0.63, 0.03; Figure 3), and by those with high negative anticipated affective reactions (*B*: −0.73; *95%CI*: 0.16, 1.36; Figure 3).

For message-induced calm, there was a significant interaction between message condition and positive anticipated affective reactions (*B*: 0.66; *95%CI*: 0.14, 1.18; Figure 3). As above, non-gain messages induced more calm than loss messages in participants with low positive anticipated affective reactions.

However, all the above effects of participants’ anticipated affective reactions did not moderate the mediating effects of the emotional processing on their affective attitude at T2.

In conclusion, even if participants experienced different emotions in response to non-gain and loss messages according to their anticipation of positive or negative emotions, this did not affect their affective attitude at Time 2. Thus, non-gain messages were more effective than loss messages, regardless of both the emotional processing activated by the messages and the receivers’ anticipated affective reactions before message exposure.

## Discussion

The results of the present study clarify the affective mechanisms triggered by differently framed messages promoting physical activity. While gain messages presented the positive outcomes deriving from doing physical activity, non-loss messages informed receivers about the avoidance of negative consequences through regular physical activity, non-gain messages provided information about the missed positive outcomes connected with low physical activity, and loss messages focused on the negative health outcomes connected with low physical activity.

What we found offers two main contributions to research on framing effects in a communication aimed at promoting physical activity. The first contribution regards the confirmation of the great impact of prefactual messages focusing on anticipated gains. In the current study, we replied to our **RQ1** showing that a positive affective attitude toward physical activity increased more after reading gain or non-gain messages than after reading loss messages or no message at all. The effectiveness of gain messages in the promotion of physical activity had been already demonstrated in previous studies (Jones et al., 2003; van’t Riet et al., 2010; Kozak et al., 2013; Williamson et al., 2020). However, for the first time in the present study we showed that also non-gain messages are effective in promoting physical activity. This result suggests that the effectiveness of message framing in the context of physical activity does not lie so much in the positive (gain or non-loss) or negative (non-gain or loss) valence of the message. It is more related to the fact of referring to the presence or absence of gains (gain and non-gain messages) rather than to the presence or absence of losses (loss and non-loss messages). Even if they have a negative valence, non-gain messages are indeed characterized by a gain-related outcome type, similar to gain messages.

It should be noted that the above outcome of message framing differs from what we previously observed when comparing the four frames in the case of home-based physical activity during the Covid-19 lockdown (Carfora and Catellani, 2021). In that study, we found that non-loss was the highest persuasive message frame. The diversity of the results is likely to be due to the social context in which the first data were collected. During the Covid-19 lockdown, people were motivated to do physical activity at home mainly to avoid losing their physical shape, which was heavily threatened by the obliged segregation at home. So, the differences in the results could be attributed to the fact that in the present study, we referred to physical activity in general and not to physical activity at home. Future studies will therefore have to take all these aspects into account and consider whether the framing effect has a different effect based on the specific type of recommendation. In the case of eating behaviors, recent studies have indicated that the effectiveness of the framing effect depends on the recommended food choice (e.g., reducing red meat consumption vs. increasing vegetable consumption; Carfora et al., 2022a;b). Similarly, in the case of sustainable behaviors, the framing effect appeared to vary according to the recommendation of reducing gas emissions or increasing renewable energy (Bertolotti and Catellani, 2014). It would therefore be useful to investigate whether this also concerns the type of physical activity recommended (such as walking, playing sports, climbing stairs, training at home, training outdoors, etc.).

The second contribution of our results regards their highlighting three key roles played by the affective variables in determining the effects of recommendation messages. First, our study showed that after exposure to the recommendation messages the affective attitude toward physical activity changed, whereas the cognitive attitude did not (disconfirming our **H1a**). Thus, we confirmed our **H1b**, related to the positive effects of prefactual messages on receivers’ affective attitudes toward physical activity, while we disconfirmed our **H1a**, related to the effect of prefactual messages on receivers’ cognitive attitudes. Moreover, changes in affective attitude fully mediated the effect of the intervention on participants’ self-reported behavior and intention, supporting our **H2a** and **H2b**. These results are consistent with past findings showing that affective attitude toward physical activity is a stronger predictor of future behavior than cognitive attitude (Rhodes et al., 2009, 2018, 2019; McEachan et al., 2011, 2016; Phipps et al., 2021). As suggested by Phipps et al. (2021), a potential explanation of this effect may stem from the primal emotional drive system of approach/avoid reactions favoring hedonistically pleasing behaviors (Cabanac, 1992; Williams, 2019). Another explanation may be grounded on the temporal discounting effect (Story et al., 2014), that is, an individual’s tendency to make unhealthy choices due to their delayed consequences. Both of these mechanisms might indeed place a greater emphasis on achieving an enjoyable hedonistic state than improved health, which is likely to be more instrumental in nature.

A second key role of affective variables when promoting physical activity is played by anticipated emotions before exposure to a messaging intervention. To address our **RQ2**, we explored whether the mediating effect of affective attitude at T2 was influenced by prior participants’ affective reactions. We showed that gain messages were especially effective in increasing a positive affective attitude and then physical activity when participants had low anticipated positive emotions before message exposure. This result suggests that when people have low anticipated positive emotions motivating them to do physical activity for obtaining gains is especially advisable. This is also in line with the empirical findings on the affective forecasting bias (Ruby et al., 2011; Loehr and Baldwin, 2014), according to which people tend to underestimate how positive physical activity will be, particularly when they rarely engage in physical activity (Loehr and Baldwin, 2014). Therefore, gain messages are a promising point of attack for interventions targeting such specific segment of the population.

The third key role of the affective variables when promoting physical activity is the one played by the emotions triggered by the recommendation messages. Consistent with previous findings (Nabi et al., 2020), we found that gain messages induce more positive and less negative emotions than loss messages (supporting our **H3**). We also found that gain messages activate greater hope. So far, only a few correlational studies have investigated and confirmed the role of hope in determining physical activity (Nothwehr et al., 2013; Anderson and Feldman, 2020). Nonetheless, hope is likely to be an important influential determinant of physical activity because it emphasizes planning and motivation (Snyder et al., 1991). Indeed, our findings show gain messages activate hope, very likely because they induce the perception that the desired goals can be met. Moreover, we found that non-gain and non-loss messages induce different emotions (answering our **RQ3**). Non-gain messages induce more anger than gain messages and non-loss messages induce less fear than loss messages. Finally, we investigated if participants experienced different emotions in response to diverse messages according to their anticipation of positive or negative emotions (**RQ4**). We found that, even if diverse levels of negative and positive anticipated affective reactions influenced how participants’ emotionally evaluated the messages, this did not influence the impact of a change in affective attitude on participants’ intention and physical activity after the intervention.

### Limitations and future directions

Our research has several limitations. First, our sample was restricted to Italian people, thus the data may not be generalized to other countries. Second, we cannot exclude the risk of self-selection bias, as participants were invited for a study on public communication. Third, participants were asked to complete the Time 2 questionnaire immediately after the end of the messaging intervention, which lasted only 14 days. Thus, we were able to assess only short-term effects. Messages delivered over a longer time span and repeated measurements of their effects could yield larger and long-term effects on recipients’ attitudes behaviors and future intentions. Fourth, we measured self-reported behavior using an online questionnaire. Self-report measures have numerous limitations, which future research can bypass by using more precise measurements of physical activity, for example by using pedometers. In sum, future research should carefully retest our preliminary results on the mechanisms involved in processing messages on physical activity formulated with different frames. Future studies could also deepen our understanding of the effects of the four types of message frames investigated here, considering their fit with other individual characteristics, such as health motives (Carfora et al., 2022b). Once said that, the results of the present study have some useful implications about how scholars, institutions, and social marketing can select message framing in their communication to enhance physical activity.

The avoidance of loss-framed messages can be deemed to be a very promising intervention. We found that such a relatively simple and low-cost intervention can lead to a significant increase in self-reported physical activity and future intention to do it. Thus, the practical implications of our results include the possibility to use these messages to promote physical activity in Italy. For example, they may be used to deliver recommendations *via* online communication within promotion campaigns. Institutions might adopt automatic chatbots for sending messages, as we did in the present study, to prompt physical activity and reduce sedentary lifestyles.

## Conclusion

In conclusion, our study contributes to explaining the effects of message frames based on the presence/absence of positive/negative outcomes of expected behaviors and aimed at changing the attitudes, intentions, and behaviors of the receivers. The current findings, together with those of previous research, suggest that physical activity is motivated more by obtaining/not losing future gains than by encountering/avoiding losses. Importantly, our results emphasize the relevant role of people’s affective evaluations in three ways. First, changing affective attitudes, rather than cognitive attitudes, is a key to promoting physical activity. Second, individual predispositions in anticipating positive emotions interact with the effectiveness of message framing. People who do not anticipate positive affective reactions toward doing physical activity are more sensitive to messages suggesting future gains. Third, this paper paves the way for future studies on the importance of formulating gain messages that can activate hope in the audience. It will be up to future research to further investigate the possibility of applying messages such as the ones employed here in contexts different from the one investigated here, as well as verifying if and how the differences in the mechanisms studied here also depend on further individual differences among receivers.

## Data availability statement

The raw data supporting the conclusions of this article will be made available by the authors, without undue reservation.

## Ethics statement

The studies involving human participants were reviewed and approved by the Scientific Committee of the Catholic University of the Sacred Heart (Milan) and the Scientific Committee of the Istituti Clinici Scientifici Maugeri (Pavia). The patients/participants provided their written informed consent to participate in this study.

## Author contributions

VC proposed research questions, planned research, analyzed the data design, wrote the paper, and took responsibility for data collection and manuscript. MB helped contributed to data analysis and paper writing. PC supervised the conception, research design, and interpretation of data and thoroughly revised this manuscript as regards contents and style. All authors contributed to the article and approved the submitted version.

## Funding

This study received funding from the project Re-HUB-ility: Rehabilitative Personalized Home System and Virtual Coaching for Chronic Treatment in Elderly, supported by Call HUB Ricerca e Innovazione by Regione Lombardia, Istituti Clinici Scientifici Maugeri, and Athics s.r.l. (Grant Number: D.G.R. N. 727 of 5/11/2018; decreto 18854 of 14/12/2018). Athics s.r.l. was not involved in the study design, collection, analysis, interpretation of data, the writing of this article or the decision to submit it for publication.

## Conflict of interest

The authors declare that the research was conducted in the absence of any commercial or financial relationships that could be construed as a potential conflict of interest.

## Publisher’s note

All claims expressed in this article are solely those of the authors and do not necessarily represent those of their affiliated organizations, or those of the publisher, the editors and the reviewers. Any product that may be evaluated in this article, or claim that may be made by its manufacturer, is not guaranteed or endorsed by the publisher.

## Footnotes

^1^PsyMe is a free smartphone app of the Catholic University of the Sacred Heart, Milan. It was developed to support scientific research in the field of social psychology and artificial intelligence (https://apps.apple.com/it/app/psyme/id1536587889; https://play.google.com/store/apps/details?id=uncatt.unipv.xtream.psyme&hl=en_US&gl=US). The PsyMe app respects participants’ privacy and anonymity thanks to the assignment of an anonymous code to each participant. It allows sending questionnaires, messages, and push notifications to remind message reading.
