# Supplementary material for: Affective components in promoting physical activity: A randomized controlled trial of message framing
Source: Front Psychol. 2022 Sep 12;13:968109. doi: 10.3389/fpsyg.2022.968109 (PMC9512085; doi:10.3389/fpsyg.2022.968109)
Supplement: Supplementary file 1 [file Table_1.docx]

**Supplementary Table 1.** *Messages Delivered in the Four Message Conditions.*

| **Gain Messages** | **Non-Loss Messages** | **Non-Gain Messages** | **Loss Messages** |
| --- | --- | --- | --- |
| If you exercise regularly,  you will improve the functioning of your cardiovascular system. | If you exercise regularly, you will avoid  worsening the functioning of your cardiovascular system. | If you do not exercise regularly,  you will miss the chance to improve the functioning of your cardiovascular system. | If you do not exercise regularly,  you will worsen the functioning of your cardiovascular system. |
| If you exercise regularly, you will improve your fitness. | If you exercise regularly, will avoid worsening your fitness. | If you do not exercise regularly,  you will miss the opportunity to improve your fitness. | If you do not exercise regularly, you  will worsen your fitness. |
| If you exercise regularly,  you will enhance your muscle strength. | If you exercise regularly, you will avoid diminishing muscle strength. | If you do not exercise regularly,  you will miss the chance to improve your muscle strength. | If you do not exercise regularly,  you will diminish your muscle strength. |
| If you exercise regularly, you will feel more agile. | If you exercise regularly, you will avoid feeling less agile. | If you do not exercise regularly,  you will miss the chance to feel more agile. | If you do not exercise regularly,  you will feel less agile. |
| If you exercise regularly, you could improve your sex life. | If you exercise regularly, you could avoid making your sex life worse. | If you do not exercise regularly,  you may miss the chance to improve your sex life. | If you do not exercise regularly,  you could make your sex life worse. |
| If you exercise regularly,  you will increase your good mood. | If you exercise regularly, you will avoid reducing your good mood. | If you do not exercise regularly,  you will lose the chance to increase your good mood. | If you do not exercise regularly,  you will reduce your good mood. |
| If you exercise regularly, you will increase your emotional well-being. | If you exercise regularly, you will avoid reducing your emotional well-being. | If you do not exercise regularly,  you will miss the opportunity to increase your emotional well-being. | If you do not exercise regularly,  you will reduce your emotional well-being. |
| If you exercise regularly, you will increase your feeling of vitality. | If you exercise regularly, you will avoid diminishing your feeling of vitality. | If you do not exercise regularly,  you will lose the opportunity to increase your feeling of vitality. | If you do not exercise regularly,  you will decrease your feeling of vitality. |
| If you exercise regularly, you will feel more in harmony with your body. | If you exercise regularly, you will avoid feeling less in harmony with your body. | If you do not exercise regularly,  you will lose the opportunity to feel more in harmony with your body. | If you do not exercise regularly,  you will feel less in harmony with your body. |
| If you exercise regularly, you will feel more satisfied. | If you exercise regularly, you will avoid feeling more dissatisfied. | If you do not exercise regularly,  you will lose the opportunity to feel more satisfied. | If you do not exercise regularly,  you will feel more dissatisfied. |
| If you exercise regularly, you will increase your well-being when you are with other people. | If you exercise regularly, you will avoid diminishing your well-being when you are with other people. | If you do not exercise regularly,  you will lose the ability to increase your well-being when you are with other people. | If you do not exercise regularly,  you will decrease your well-being when you are with other people. |
| If you exercise regularly, you will be more appreciated by others. | If you exercise regularly, you will avoid being less appreciated by others. | If you do not exercise regularly,  you will lose the opportunity to be more appreciated by others. | If you do not exercise regularly,  you will be less appreciated by others. |
| If you exercise regularly, you will feel more comfortable with other people. | If you exercise regularly, you will avoid feeling less comfortable with other people. | If you do not exercise regularly,  you will lose the opportunity to feel more comfortable with other people. | If you do not exercise regularly,  you will feel less comfortable with other people. |
| If you exercise regularly, you will feel more approved by other people. | If you exercise regularly, you will avoid feeling less approved by other people. | If you do not exercise regularly,  you will lose the opportunity to feel more approved by other people. | If you do not exercise regularly,  you will feel less approved by other people. |
| If you exercise regularly, you will increase your independence on others. | If you exercise regularly, you will avoid increasing your dependence on others. | If you do not exercise regularly,  you will miss the chance to increase your independence on others. | If you do not exercise regularly,  you will increase your dependence on others. |
